# Whole Brain Expression of Bipolar Disorder Associated Genes: Structural and Genetic Analyses

**DOI:** 10.1371/journal.pone.0100204

**Published:** 2014-06-18

**Authors:** Michael J. McCarthy, Sherri Liang, Andrea D. Spadoni, John R. Kelsoe, Alan N. Simmons

**Affiliations:** 1 Research Service, Veterans Affairs San Diego Healthcare System, San Diego, California, United States of America; 2 Department of Psychiatry, University of California San Diego, La Jolla, California, United States of America; University of Iowa Hospitals & Clinics, United States of America

## Abstract

Studies of bipolar disorder (BD) suggest a genetic basis of the illness that alters brain function and morphology. In recent years, a number of genetic variants associated with BD have been identified. However, little is known about the associated genes, or brain circuits that rely upon their function. Using an anatomically comprehensive survey of the human transcriptome (The Allen Brain Atlas), we mapped the expression of 58 genes with suspected involvement in BD based upon their relationship to SNPs identified in genome wide association studies (GWAS). We then conducted a meta-analysis of structural MRI studies to identify brain regions that are abnormal in BD. Of 58 BD associated genes, 22 had anatomically distinct expression patterns that could be categorized into one of three clusters (C1–C3). Brain regions with the highest and lowest expression of these genes did not overlap strongly with anatomical sites identified as abnormal by structural MRI except in the parahippocampal gyrus, the inferior/superior temporal gyrus and the cerebellar vermis, regions where overlap was significant. Using the 22 genes in C1–C3 as reference points, additional genes with correlated expression patterns were identified and organized into sets based on similarity. Further analysis revealed that five of these gene sets were significantly associated with BD, suggesting that anatomical expression profile is correlated with genetic susceptibility to BD, particularly for genes in C2. Our data suggest that expression profiles of BD-associated genes do not explain the majority of structural abnormalities observed in BD, but may be useful in identifying new candidate genes. Our results highlight the complex neuroanatomical basis of BD, and reinforce illness models that emphasize impaired brain connectivity.

## Introduction

Bipolar disorder (BD) is a severe mental illness typified by depression, mania, psychosis and neurocognitive deficits. Based upon family and twin studies, a genetic basis of the illness is strongly suspected [1], but the genes responsible for BD remain largely unknown. Large meta-analyses of genome-wide association studies (GWAS) of BD have been conducted, identifying risk alleles at *ODZ4, ANK3, CACNA1C,* and *ITIH3* and others with weaker, but suggestive associations [2]. The evidence collected from these and other studies indicate that BD is genetically complex, with no single genetic locus explaining more than a small portion of the variance in BD. Nonetheless, approximately 25% of the genetic variance underlying BD can be explained by common gene variation, using polygenic models [3].

The underlying assumption guiding psychiatric genetics is that variation in genes ultimately affects neural pathways in the brain to cause illness. Attempts to organize genes into pathways, and deduce their function often focus on strategies based upon gene ontology (GO). However, these methods often say little about neuroanatomical context, or the tissue specific functions of a particular gene, making GO potentially susceptible to artifacts, and misleading conclusions. For instance, two calcium channel genes may cluster based upon overlap in their predicted functions, but one could be expressed only in the heart, while the other is restricted to neurons, thereby undermining the physiological significance of their association. For this reason, while some success has been reported in the use of pathways as an organizational framework for BD [4], little is known about candidate genes in BD in terms of their actual distribution in the nervous system and the capacity for biologically meaningful interaction among risk-associated genes.

From the structural perspective, brain imaging studies in BD have described changes in cortical thickness [5], and cortical volume [6], [7], but have not yet been able to formulate these findings in terms of a comprehensive understanding of the illness based on specific genetic or molecular mechanisms. While a number of studies have examined the genetic basis of brain morphology in more detail [8], [9], or conducted SNP associations with anatomical features of interest [10]–[13], the majority of these studies have either examined a small number of brain areas and/or candidate genes, or have not been conducted in clinical samples. Due to these shortcomings, it is unclear how genetic factors affect brain structure, and which brain regions are most important in BD.

We have collected information from multiple sources, including a large meta-analysis of GWAS in BD [2], 18 structural MRI studies of BD [13]–[29], and a whole genome/whole brain gene expression map [30] to investigate the relationship between genes and anatomy in BD. Using the GWAS results to prioritize a set of genes with strong evidence for BD risk-association, expression patterns were examined across ∼900 brain structures, identifying three major expression clusters. Superimposing the BD-associated gene clusters onto a structural imaging map of neuroanatomical variation in BD, we tested two hypotheses. First, that BD-risk associated gene expression will be enriched in brain regions with structural alterations in BD, and second, that genes with anatomical expression patterns similar to BD-associated genes, will themselves harbor BD risk variants. Our results reveal partial support for each hypothesis, and reinforce models of BD in which genetic risk is widely, but not uniformly spread across multiple brain regions, some of which may be nodes that are susceptible to genetic variation and structure change.

## Methods

### BD-associated Gene Selection

The top 100 BD-associated SNPs from the 2011 PGC-BD meta-analysis were examined, yielding a list of SNPs with genome wide association p-values ranging from 4.7×10^−5^ to 5.5×10^−10^, corresponding to the top ∼10% of genetic variants identified in the meta-analysis of >11,000 BD cases and >51,000 controls [2]. Each SNP was examined to identify those that were associated (within 100 kb) of a single gene. Intergenic variants or SNPs in close proximity to multiple genes were excluded from further analyses. In this manner, 58 genes were selected for brain mapping (Table 1).

**Table 1:** Genes in proximity to BD-associated SNPs.

Gene Name			
ADCY2	FAM155A	MSI2	SIPA1L2
AKAP13	FLJ16124	NFIX	SNX8
ANK3	FSTL5	NGF	SPERT
ANKS1A	GATA5	NPAS3	STK39
ATP6V1G3	GNA14	ODZ4	SYNE1
ATXN1	GPR81	PAPOLG	THSD7A
C11orf80	HHAT	PAX1	TNR
C15orf53	IFI44	PBRM1	TRANK1
CACNA1C	ITIH3	PTPRE	TRIM9
CACNA1D	KDM5B	PTPRT	UBE2E3
CACNB3	KIF1A	RASIP1	UBR1
CROT	LOC150197	RIMBP2	ZMIZ1
DLG2	MAD1L1	RXRG	ZNF274
DNAJB4	MAPK10	SGCG	
DUSP22	MCM9	SH3PXD2A	

### Whole Brain, Genome Wide Expression Analysis

At the time the data were accessed (October, 2011), the Allen Brain Institute (ABI, website: http://www.brain-map.org) had complete microarray gene expression results available from two, previously healthy human male brains (age 24 and 39 yr). From these data, expression values for 58 BD-associated genes in ∼900 brain regions were selected. Overlap in the anatomical regions sampled between the brains was excellent, but for a small number of regions, only one brain sample was available. Since we first accessed the data, additional brains have become available but some of these are incomplete. For this reason, these later additions were not included in the analysis. Comprehensive brain and RNA quality control was conducted by ABI. The details of this process are available on their website. In brief, post mortem interval was 25 hr and 30 hr respectively. RNA was examined to exclude degradation, and RNA integrity values (RIN) ranged from 5.4–7.1. Brain pH >6.0 was required of all samples. Additional details of brain preparation and microarray study design were published in detail previously [30]. In all cases, at least one transcript corresponding to the gene of interest was identified, and in most cases two or more probes for the same gene were available. A total of 105 transcripts were identified. For each transcript, expression level was recorded for each anatomical region from both brains.

To identify genes with similar expression profiles, expression of each gene was analyzed by hierarchical clustering across ∼900 neuroanatomical regions, using a nearest neighbor similarity measure (StatisticXL). Data for each gene were normalized to Z-scores across brain regions in order to identify anatomical regions that are enriched or depleted in the expression of an individual gene. Average Z-scores were then determined for each cluster (C1–C3) at each anatomical site. Each of the ∼900 brain regions in the Allen Brain Atlas is associated with three dimensional X, Y, Z coordinates, which correspond to the anatomical site of origin. For improved visualization, right and left hemisphere values were averaged and regional expression blurred (FWHM = 12 mm) before being mapped to an idealized brain using Analysis of Function Images (AFNI) [31].

### Structural MRI Meta-analysis

Studies were identified with an online citation indexing service (Medline) using “brain”, “volume”, and “bipolar disorder” as search terms. This search yielded 275 articles published between 1985 and 2011. These search results were filtered to include only voxel based morphometry (VBM) studies that published volume/density results as 3D coordinates (X, Y, Z) in stereotactic space, compared the target population (BD type 1) to controls, included greater than six subjects, and did not use data already published in similar analyses. Filtering the results yielded 18 articles. The data from these were then entered into the Ginger ALE program (www.brainmap.org) to identify regions of statistical significance. Subsequently, coordinates were transformed to Talairach space for studies that had published coordinates in the Montreal Neurological Institute (MNI) space according to the nonlinear Brett transformation [32] included in the BrainMap environment, to allow analysis relative to a single template. Within GingerALE study specific Gaussian blurring was applied ranging from ∼9 to ∼8.5 mm depending on the study sample size.

Two activation likelihood estimation (ALE) maps were created as described [33]. One map displays regions with increased greater gray matter volume/density in BD compared to controls, the second displays regions with greater gray matter density/volume in controls compared to BD subjects. The data consider 1,069 BD and control subjects in total. Statistical significance was determined using a permutation test of randomly generated foci. No assumptions were made concerning the distribution or spatial separation of these random foci. Five thousand permutations were computed using the same FWHM value and the same number of foci used in computing the ALE values. The test was corrected for multiple comparisons using the false discovery rate (FDR) method [34], [35]. The minimum cluster volume required was 224 mm^3^. All data processing was carried out using a Java version of Ginger ALE (www.brainmap.org). Whole-brain maps of the ALE values were imported into MRIcroN [36] for data visualization and overlaid onto an anatomical template generated by spatially normalizing the International Consortium for Brain Mapping (ICBM) template to Talairach space [37].

### Determination of Overlap between Structural Changes and Gene Expression Clusters

The distances between foci from gene expression cluster peaks and structural abnormalities identified in the meta analysis were calculated using the following: 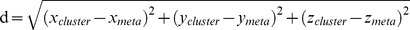. To determine a distance of statistically meaningful proximity, 200,000 random brain voxel pairs were created and a cut point for an alpha of.05,.01, and.005 were determined empirically for distances (23 mm, 13 mm, and 10 mm; respectively). While this does not necessarily imply anatomical overlap, it does suggest proximity that would be unlikely to occur by chance.

### Set-based Test of Gene Expression Profiles

Each of the clustered, BD-associated genes (index genes) was analyzed using the Allen Brain Atlas Neuroblast function [38] to generate a rank ordered list of genes that correlate in expression to the index gene. In this way, genes whose expression is highly correlated with the index can be readily identified and arranged in descending order by correlation coefficient. For each index gene, the top 10 and top 25 most similarly expressed genes were identified, and defined as a set. Data were filtered so that redundant probes or probes that did not correspond to a named human transcript were discarded. No correlation coefficient threshold was employed for set inclusion, but in all cases correlation with the index gene was high (mean r = 0.68, range 0.50–0.94). A set based genetic association analysis was conducted in PLINK, using genome-wide SNP data from 2200 Caucasian BD cases and 1436 controls. BD subjects were ascertained and genotyped as two independent cohorts for GWAS studies of BD that have been described in detail previously [39], [40]. Control subjects have also been described in detail previously [41]. For each gene, all SNPs for which genotype data exist were included in the set-based analysis. SNPs were then filtered using PLINK to consider only informative markers (based on linkage disequilibrium, defined as r^2^≥0.50). No maximum number of SNPs was specified. After SNPs in were removed, variants in genes sets were examined to determine their association with BD. In these analyses, each set was analyzed using the genes with the top 10, and 25 (including index) most similar expression profiles. In addition, to avoid circular arguments in which index genes conferred association to the expression-based set, each analysis was repeated with the index gene removed (top 24) to ensure association of the set independently of the index gene. Each set and level of inclusion was run over 10,000 permutations. In all, 22 sets were run, each with three levels. Statistical significance was defined when p<0.05, and trend level significance was defined when p<0.10.

## Results

### Unsupervised Gene Clustering

**Figure 1:**
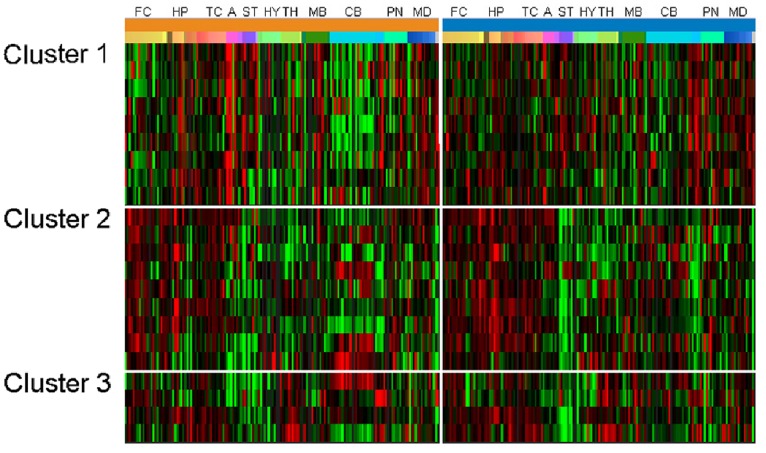
Whole brain expression patterns of BD-associated genes. Expression of 22 BD-associated genes is organized by cluster for each of two brains (indicated by the thick orange and blue line). Each row represents a single gene, and each column represents one of ∼900 brain regions. Brain regions are organized anatomically, by broadly defined regions where FC: frontal cortex, HP: hippocampus, TC: temporal cortex, A: amygdala ST: striatum, HY: hypothalamus, TH: thalamus, MB: midbrain, CB: cerebellum, PN: pontine nuclei, MD: medulla.

If BD preferentially alters function in specific brain structures, some BD-associated genes may be similarly expressed in patterns that include the affected regions. To determine if this was the case, we first examined the anatomical expression profile of all 58 BD-associated genes to identify common patterns of high/low expression across the brain (Table 1). Three reproducible gene expression clusters were identified, accounting for 22 genes. A fourth set of six genes reliably failed to cluster. The former sets were termed clusters 1, 2 and 3 (C1, C2, C3) and used for subsequent analysis (Figure 1). The latter set was not considered further. Within a cluster, gene expression values had pair wise correlation coefficients of 0.4–0.8, indicating moderate-high correlation across brain regions. In some cases, correlation across clusters was noted, particularly between C2 and C3. This correlation was typically less than 0.2 and always less than the correlation within the cluster. Gene cluster assignments are indicated in Table 2.

**Table 2:** Bipolar disorder associated gene expression clusters.

Cluster 1	Cluster 2	Cluster 3
ATP6V1G3	ADCY2	ANK3
GATA5	CACNB3	STK39
GPR81	DLG2	FAM155A
NGF	KDM5B	SIPA1L2
NPAS3	KIF1A	
PAX1	MAPK10	
SPERT	RIMBP2	
TNR	UBE2E3	
ZMIZ1	UBR1	

### Brain Mapping of Gene Clusters

**Figure 2:**
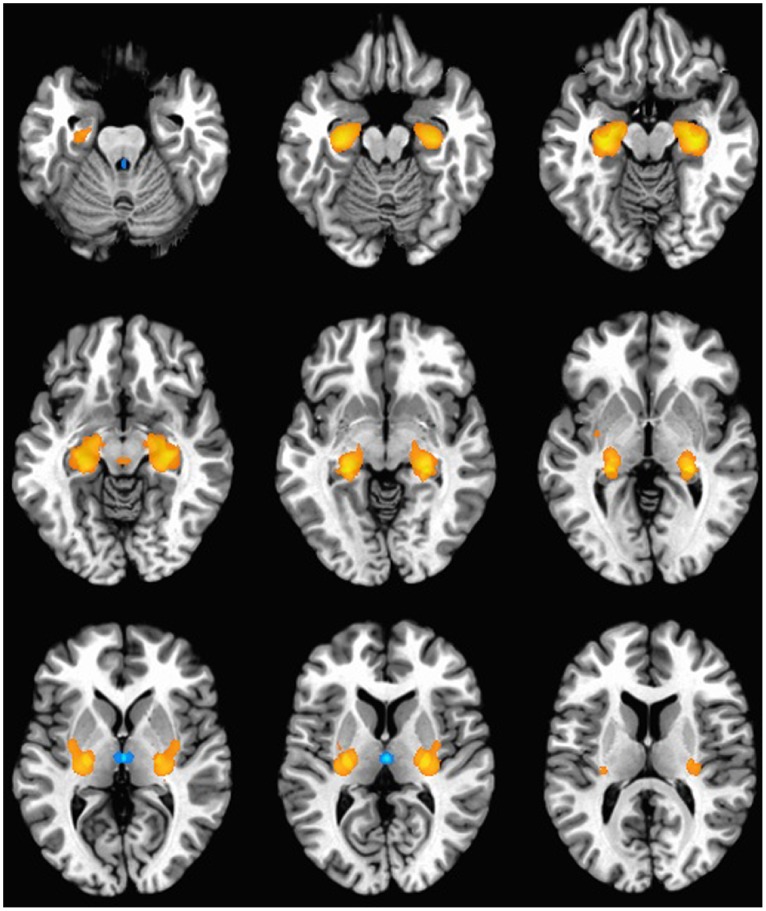
Cluster 1 gene expression pattern. An idealized human brain shown in horizontal cross section indicates the anatomical regions enriched (orange and red) and depleted (blue) in gene expression associated with C1. For improved visualization, gene expression in corresponding regions of the right and left hemispheres has been consolidated and shown in mirror image bilaterally. C1 is defined by high expression in the parahippocampal gyrus, hippocampus and posterior thalamus.

**Figure 3:**
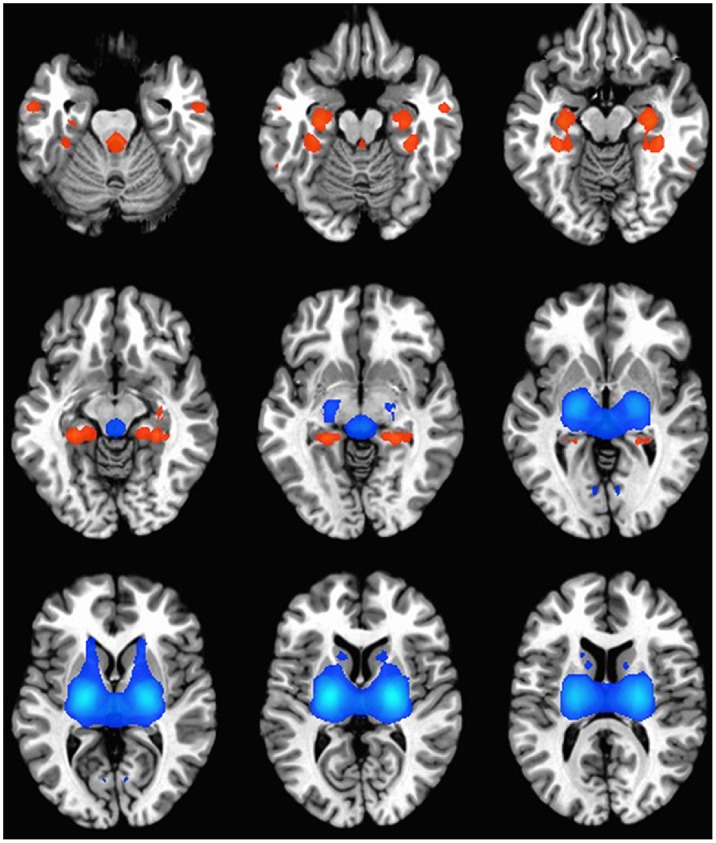
Cluster 2 gene expression pattern. An idealized human brain shown in horizontal cross section indicates the anatomical regions enriched (orange and red) and depleted (blue) in gene expression associated with C2. For improved visualization, gene expression in corresponding regions of the right and left hemispheres has been consolidated and shown in mirror image bilaterally. C2 is defined by high expression in portions of the hippocampus, temporal cortex, and midbrain, and very low expression in the striatum, and posterior thalamus.

**Figure 4:**
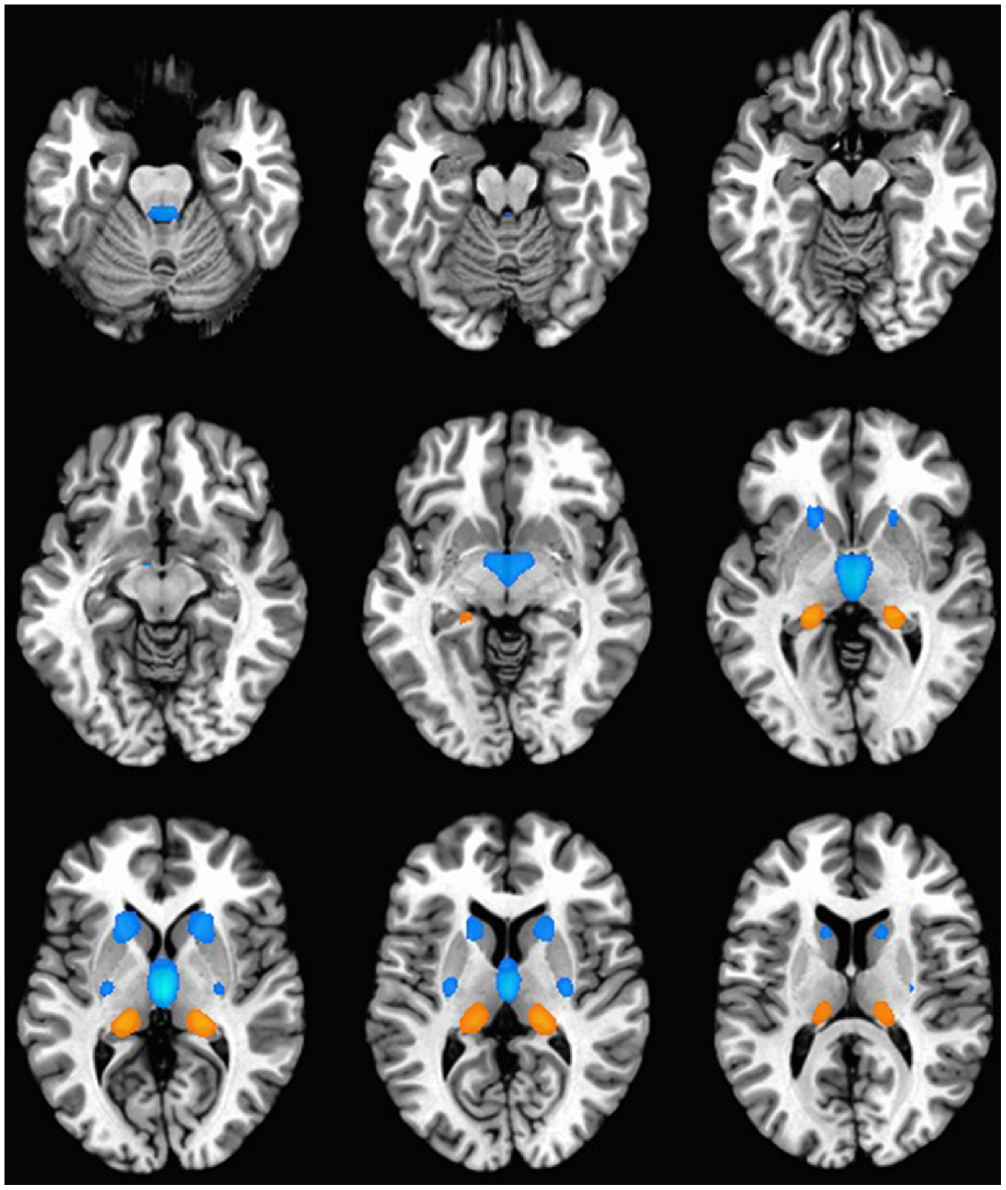
Cluster 3 gene expression pattern. An idealized human brain shown in horizontal cross section indicates the anatomical regions enriched (orange and red) and depleted (blue) in gene expression associated with C3. For improved visualization, gene expression in corresponding regions of the right and left hemispheres has been consolidated and shown in mirror image bilaterally. C3 is defined by high expression in the posterior thalamus and very low expression in the striatum (caudate and putamen).

In order to map common anatomical patterns of gene expression in three dimensions, and identify areas of relative enrichment or depletion, the average expression data for regions of minimum and maximum expression for each cluster were projected onto an idealized brain**.** All three clusters were expressed widely across multiple brain regions, but few regions met statistical cut-offs for inclusion in mapping. Visualization of the expression patterns of the three clusters revealed that C1 was defined by high expression in the parahippocampal gyrus, hippocampus and posterior thalamus (Figure 2). C2 was expressed highly in portions of the hippocampus, temporal cortex, and midbrain, and expressed at very low levels in the striatum, and posterior thalamus (Figure 3). C3 was expressed highly in the posterior thalamus and was expressed at very low levels in the caudate, and putamen (Figure 4).

### Structural Differences in Bipolar vs. Control Brains

**Figure 5:**
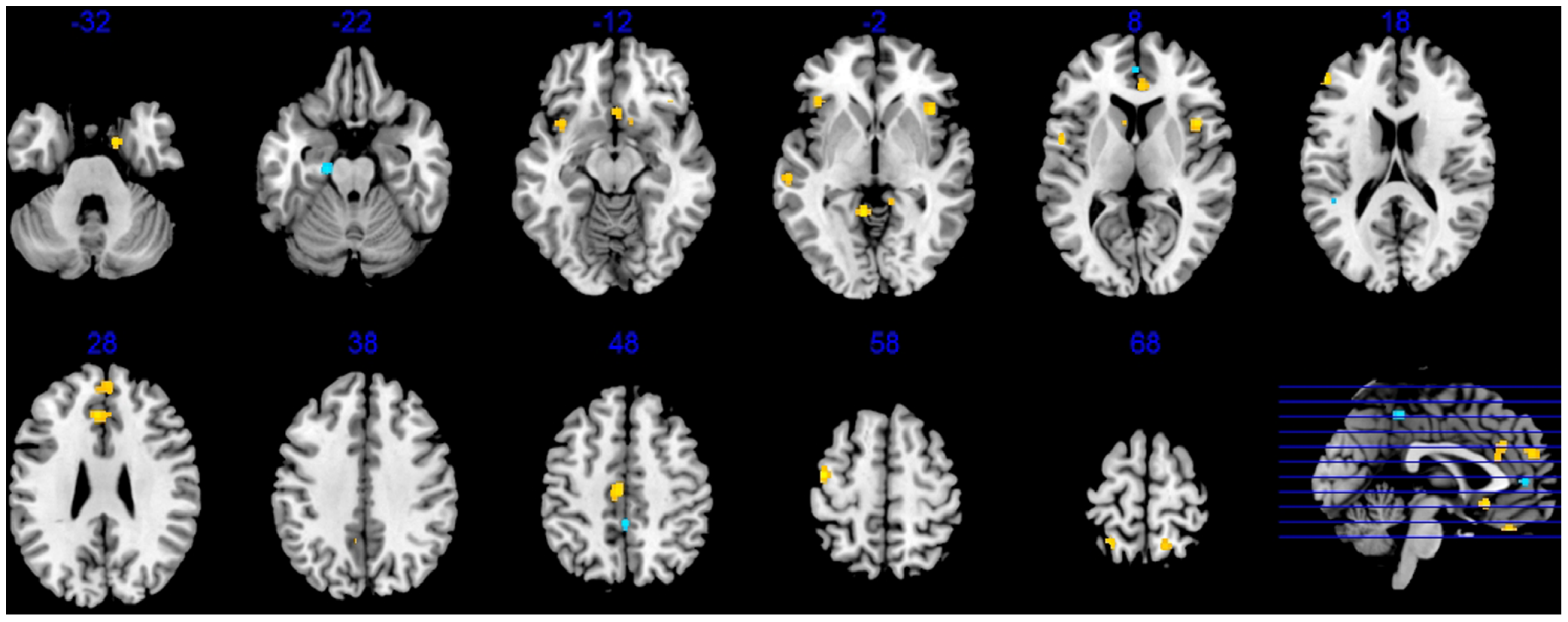
Brain regions identified by volumetric meta analysis. An idealized human brain shown in horizontal cross section indicates the anatomical regions that are on average, larger in volume in controls compared to BD (yellow) or smaller in controls compared to BD (blue).

A meta-analysis of structural MRI studies was conducted to identify the most reliably affected brain regions in BD. Twenty three regions were identified that differed in density/volume between BD cases and healthy controls (Figure 5, Table 3). Twenty of these regions indicated greater gray matter density/volume in controls compared to BD subjects. Thirteen of the regions identified were in the frontal/cingulate/insula cortex, and six were located in the temporal and cortical regions. The remainder was distributed in the cerebellum and parietal cortex. Three left sided regions (cingulate gyrus, parahippocampal gyrus, paracentral lobule) were larger in BD subjects compared to controls.

**Table 3:** Meta analysis results for anatomical regions with differential volume.

CON>BD					
Volume (mm^3^)	x	y	z	Location	ALE Value
1064	−3	31	27	Left cingulate gyrus	0.019
776	−5	−19	49	Left medial frontal gyrus	0.020
696	3	52	25	Right medial frontal gyrus	0.019
664	53	−31	3	Right superior temporal gyrus	0.020
528	−43	−9	56	Left precentral gyrus	0.020
448	−19	−1	−37	Left uncus	0.018
448	−37	11	−13	Left inferior frontal gyrus	0.018
448	−59	−23	−3	Left middle temporal gyrus	0.018
448	5	37	11	Right anterior cingulate	0.018
432	13	−55	71	Right postcentral gyrus	0.018
408	39	21	0	Right insula	0.018
400	2	19	−9	Right subcallosal gyrus	0.018
392	43	11	9	Right insula	0.017
384	17	−1	−30	Right uncus	0.018
384	11	−41	−4	Right cerebellar vermis	0.018
384	−6	−45	−3	Left cerebellar vermis	0.018
384	−50	1	5	Left superior temporal gyrus	0.018
384	−26	−54	66	Left superior parietal lobule	0.019
360	−40	26	−5	Left inferior frontal gyrus	0.017
224	−49	40	19	Left middle frontal gyrus	0.018
**BD>CON**					
**Volume (mm^3^)**	**x**	**y**	**z**	**Location**	**ALE Value**
304	−4	−33	33	Left cingulate gyrus	0.018
296	−19	−18	−21	Left parahippocampal gyrus	0.016
256	0	−39	50	Left paracentral lobule	0.017

### Anatomical Comparison of Gene Expression Clusters to Structural Imaging Results

The reason for regional brain volume alteration in BD is unknown**,** possibly reflecting genetic predisposition, developmental abnormality and/or pathological features of illness progression. Using the results of our meta-analysis to define the anatomically affected brain areas, we tested the hypothesis that brain volume alterations in BD are primarily genetic, looking for overlap across anatomical regions that are enriched or depleted in BD-associated gene expression and those that differ in structural morphology in BD compared to controls. On the whole the overlap was modest. In general, gene expression peaks were identified primarily in sub-cortical structures whereas neuroanatomical differences were localized in the frontal and temporal cortexes. However, there were exceptions. The parahippocampal gyrus, was enriched in both C1 and C2 expression and showed evidence of increased volume among BD patients; a region spanning the inferior/superior temporal gyrus with volume differences in BD overlapped with C2 expression; and a region of the cerebellar vermis was in close proximity to expression peak of both C2 and C3 (Table 4).

**Table 4:** Brain region overlap between gene expression clustering and differential volume.

MRI Region	Gene Expression Peak	Significance Level	Cluster
parahippocampal gyrus	parahippocampal gyrus	p<0.005	1
parahippocampal gyrus	parahippocampal gyrus	p<0.01	2
cerebellar vermis	cerebellar vermis	p<0.05	2
superior temporal gyrus	inferior temporal gyrus	p<0.05	2
cerebellar vermis	cerebellar vermis	p<0.05	3

### Gene Expression Pattern Predicts Genetic Risk-association for Bipolar Disorder

**Figure 6:**
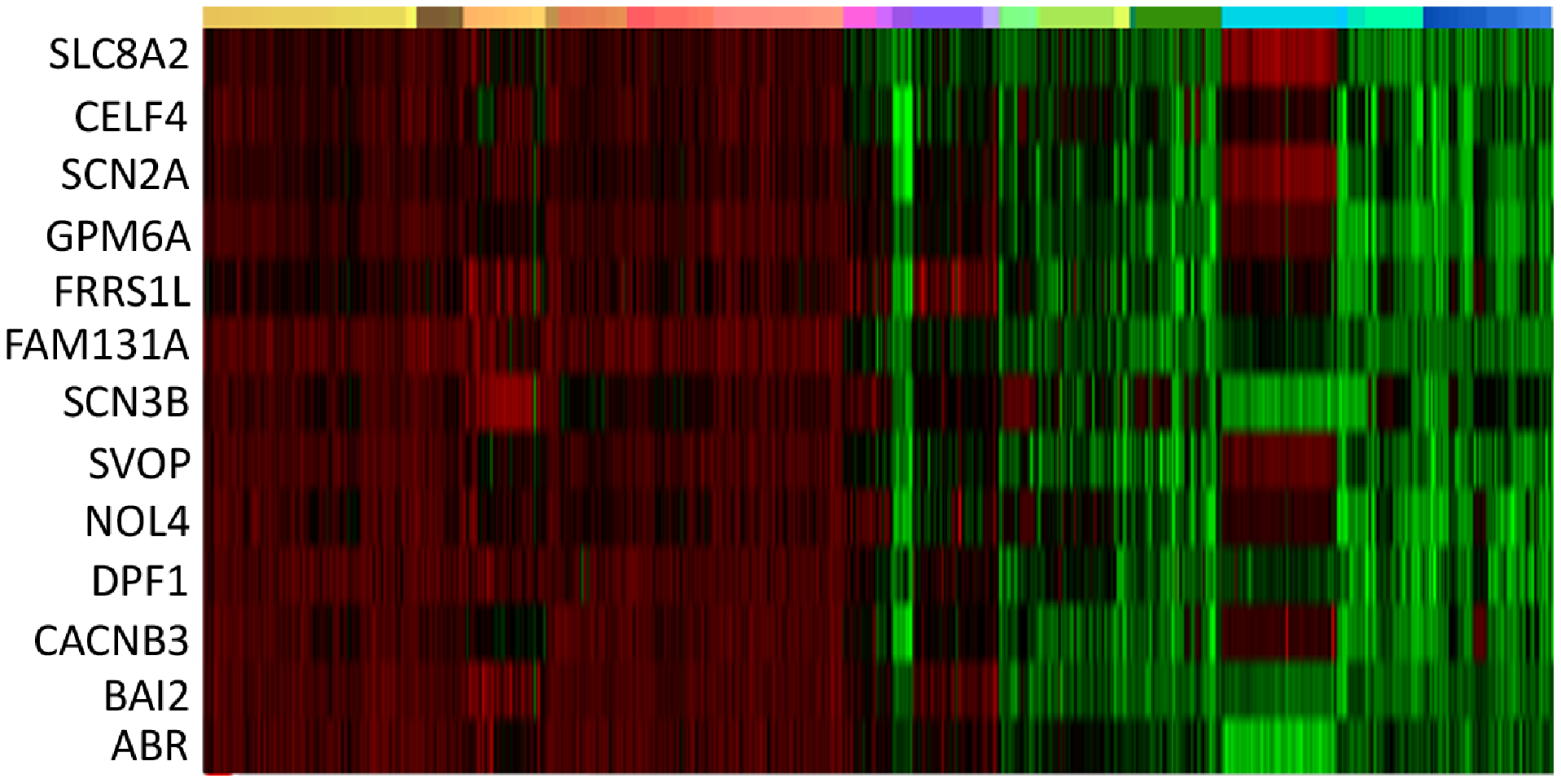
Gene expression pattern of CACNB3 is correlated with BD risk associated genes identified by set analysis. Genes that are expressed in anatomical patterns similar to BD-associated genes are more likely to contain risk-associated SNPs in set based genetic analyses. The C2 gene, *CACNB3* is shown as an example, in which twelve additional genes with expression patterns highly correlated with *CACNB3* are shown. The same genes are in close proximity to, or contain SNPs that contributed signal to the set-based association with BD, even though they were not previously identified as having strong associations in GWAS. Brain regions are organized anatomically, FC: frontal cortex, IN: insula, CN: cingulate, HP: hippocampus, L: lingual gyrus, TC: temporal cortex, A: amygdale, ST: striatum, HY: hypothalamus, TH: thalamus, MB: midbrain, CB: cerebellum, PN: pontine nuclei, MD: medulla.

It has been reported that genes expressed with similar anatomical patterns share functional similarity [30]. In our study, we hypothesized that genes with similar expression patterns share BD risk susceptibility by affecting overlapping brain circuits. We tested this idea by looking for evidence of genetic association with BD among genes that are expressed in patterns similar to genes that were identified previously as harboring risk associated alleles. Each of the 22 BD-associated genes in C1–C3 was designated as an “index”, and used for a reference against which genes with highly similar expression patterns were identified using NeuroBlast [38]. Genes expressed similarly to the index were termed “sets”. The complete list of genes in each set is shown in Table S1. Of 22 sets used for set-based genetic analysis, five showed significant evidence of BD risk-association (sets based on *ATP6V1G3*, *ADCY2, CACNB3*, *RIMBP2*, and *UBR1* as the indexes, see example Figure 6). Two other sets demonstrated trend-level evidence of association (sets based on *NGF, ZMIZ1*). In two cases (*RIMBP2*, *ATP6V1G3*), removal of the index gene substantially reduced the strength of the association, suggesting the index gene was largely responsible for conferring the BD risk associated with the set (compare top 24 vs. top 25, Table 5). However, in the remaining cases, gene set associations remained significant after removing the index gene, indicating that significant genetic risk is conferred independently by the remaining genes in the set. An example is shown in Figure 6.

**Table 5:** Set-based analyses of genes sharing expression profile with C1–C3.

Cluster	Index Gene	Top 10	Top 24	Top 25	Mean Correlation
1	**ATP6V1G3** *	**0.02**	0.98	**0.10**	0.76
1	GATA5	0.14	0.74	0.21	0.66
1	GPR81	0.92	0.78	0.83	0.79
1	**NGF** **	0.53	**0.10**	**0.09**	0.49
1	NPAS3	0.21	0.16	0.22	0.91
1	PAX1	0.17	0.67	0.53	0.48
1	SPERT	0.34	0.91	0.79	0.81
1	TNR	0.57	0.55	0.41	0.40
1	**ZMIZ1** **	0.29	**0.08**	**0.10**	0.51
2	**ADCY2** *	**0.02**	**0.02**	0.12	0.80
2	**CACNB3** *	0.18	**0.04**	0.12	0.75
2	DLG2	0.14	0.48	0.26	0.58
2	KDM5B	0.88	0.96	0.96	0.57
2	KIF1A	0.29	0.24	0.16	0.80
2	MAPK10	0.81	0.58	0.69	0.80
2	**RIMBP2** *	**0.07**	0.21	**0.04**	0.76
2	UBE2E3	0.85	0.61	0.79	0.73
2	**UBR1** *	**0.03**	**0.05**	**0.08**	0.75
3	ANK3	0.69	0.63	0.72	0.64
3	FAM155A	0.67	0.69	0.59	0.70
3	SIPA1L2	0.76	0.49	0.65	0.63
3	STK39	0.43	0.36	0.29	0.68

*significant set-based association.

**trend towards set-based association.

There was no relationship between the strength of the SNP’s BD-association in the index gene, and the subsequent set-based association. That is to say, SNPs strongly associated with BD did not necessarily make better index genes, and did not predict the BD association of expression based sets.

However, index gene clusters were not represented evenly among the enriched sets. Four out of five sets came from genes in C2, and none came from C3. Since cluster designation is organized around similarity in gene expression patterns, it is possible that overlap exists among the BD-associated gene sets (e.g. genes expressed similarly to *ADCY2*, may also be expressed like *CACNB3*). To examine whether shared genes across multiple sets contributed to any of the set-based BD-association, we examined the composition of each of the five significantly associated sets. In C1, in the set defined by *ATP6V1G3-*like expression, there was extensive overlap (11/25 genes) among sets that were not associated with BD, but no overlap with NGF-like or ZMIZ-like genes, the other C1 sets that did show suggestive BD association. Similarly, there was no overlap between the *NGF*-like and *ZMIZ*-like sets. This suggests that the genes conferring BD-risk association in C1 sets were uniquely associated with the index gene. In C2, the *ADCY2*-like and *UBR1*-like sets did not overlap with any of the other BD-associated sets. In contrast, the sets defined by *CACNB3-*like, and *RIMBP2*-like expression overlapped considerably with each other (4/25 genes), sharing the genes *ENC1*, *DPF1*, *SCN3B*, and *SVOP* in common, suggesting that the BD-risk association with these two sets may be attributable to common genetic factors.

## Discussion

Using a comprehensive, whole brain map of gene expression, we report preliminary progress in filling in the gap that exists between our understanding of the genetic and neuroanatomical bases of BD. Our results show that gene expression reproducibly classifies 38% (22/58) of the most strongly associated genes into three clusters, indicating that many BD susceptibility genes share similar expression patterns across the brain. We made two major assumptions in conducting our work. First, we assume that BD-associated variants preferentially affect genes, a position supported by data showing that BD associated SNPs are enriched in regulatory regions likely to affect gene expression and/or alter mRNA transcripts [40], [42]. A second assumption is that brain regions with the highest/lowest levels of gene expression are the most vulnerable to perturbed expression, and that under pathological conditions, these regions may have particularly severe consequences for the progression of BD. Our data suggest that at least in terms of volumetric change, this assertion may not be true. Functional differences that do not affect gray matter volume may still follow the pattern we expected, but were not assessed by our study.

### Study Limitations and Potential Confounds

Our analysis is limited by the small number of brains available for study. Furthermore, because of this small sample, it is possible that our classification scheme is influenced by artifacts from post mortem brain processing and/or RNA degradation. Because the ABI conducted careful quality control for the samples, and we selected only genes that were reliably expressed in both brain samples, we consider this risk to be small. However, replication in larger brain samples will be required to validate our expression based clustering strategies.

Instead, our meta-analysis of the structural imaging literature and other reports [6], [7], [9] support the conclusion that widespread cortical abnormalities are a key feature of BD. We have extended this finding to show that in most cases, structural brain abnormalities in BD do not overlap with regions enriched or depleted in the expression of risk-associated genes. The overlap in expression among C1/C2 genes and structural abnormalities in the hippocampus may be an important exception. Our results suggest that anatomical expression profile may be useful in identifying novel BD susceptibility genes by revealing anatomical overlap with previously detected genes associated with BD.

### Comparison with Alternate Approaches

Authors from the ABI, using the same two brains used the entire human genome to perform weighted gene co-expression network analysis (WGCNA) [30]. With this much larger number of genes, 13 gene expression modules were identified (M1-13). We compared our cluster assignments to the WGCNA-modules, and found that for the 58 bipolar-associated genes we categorized, the WGCNA module system gave similar results, but with some notable differences. For C1, 3/9 genes (33%) were mapped to M4, two were mapped to M3 (22%), one was mapped to M2 (11%), while the C1 gene *GPR81* was not assigned to any module. For C2, 9/9 genes (100%) we assigned to M2. C3 corresponded to M1, but *FAM155A*, *STK39* also showed correlation with M2, and *SIPA1L2* was not categorized by WGCNA. We also checked the 58 BD associated genes for omissions from clusters that would be suggested using the WGCNA modules. In this way, *C15orf53* (M4) could be categorized in C1; and *CACNA1C, FSTL5, MAD1L1, RASIP1, SNX8* (all M2) could be categorized in C2. Interestingly, a new cluster would be needed to include the genes *CACNA1D, ODZ4, PAPOLG, PTPRE* and *SYNE1* that did not reliably cluster in our experiment but were all categorized as M7 using the alternative method. In general, genes that were discordant between classification methods were found to have significant cross-correlation to two or more modules, suggesting their assignment to clusters in our study did not reflect methodological error, but instead reflects genuine ambiguity in the profiles. The differences in statistical methods between studies leading to different cut-offs and subsequent group assignments may reflect differences in sample size and statistical power, but may also be to some extent arbitrary.

### Structural Brain Differences in BD

The majority of studies examining gray matter in BD individuals in relation to control subjects report widespread reductions in gray matter density and/or volume in cortical regions. Consistent with this, our meta analyses identified 20 regions of reduced gray matter volume, distributed most heavily throughout frontal regions, and only three areas of increased gray matter volume in BD individuals. This empirical summary suggests that BD individuals appear to be at greatest risk for gray matter reductions in prefrontal regions.

### Gene Expression Clusters Rarely Overlap with Structurally Abnormal Regions

We found little support for the hypothesis that brain regions enriched in illness-associated genes were the most implicated in structural imaging experiments. This may be true for at least two reasons. First, gene expression was mapped in cross-section in adult brains carefully selected for the absence of psychiatric illness. Hence, it may be that the intensity and/or distribution of gene expression is perturbed in the brains of BD subjects, but not controls; or that at earlier stages of development, gene expression transiently and strongly overlaps with the brain regions in question, but this overlap diminishes during maturation. Second, the majority of the structural differences were detected in cortical regions, while the majority of high/low gene-expressing regions were sub-cortical. Gene expression occurs in the cell nucleus, but the protein products of genes often function elsewhere the cell, including at axon terminals that terminate in other brain regions. Therefore, the observed mismatch of gene expression and structural change could reflect properties of BD that affect brain circuits, whereby aberrant gene function in sub-cortical regions affects the morphology in cortical structures indirectly, perhaps through synaptic processes or distal axonal projections, causing the anatomical changes in the illness to appear away from the primary site of gene action. Recent work indeed affirms this notion, identifying loss of white matter integrity in BD, and genetic liability for BD affecting association with matter connectivity [43], [44] supporting the idea that white matter, in addition to cortical and subcortical gray matter volume is an important substrate for genetic variation in BD.

### Gene Expression Profile Predicts Association with Bipolar Disorder

Perhaps the most important result of our present work is that anatomical expression pattern is shared across BD-risk associated genes, and can be used to identify novel BD-associated variants. The number of BD-associated index genes we studied was small, and therefore it is likely that our index genes account for only a small portion of the total genetic risk for BD. Nonetheless, we conclude that there is information pertinent to understanding the nature of the illness in this subset of genes. This was particularly true of C2 genes (*ADCY2, CACNB3, RIMBP2, UBR1*) which were enriched in correlated gene sets associated with BD. C2 corresponds to the M2 module described previously, and gene ontology (GO) analysis of this module was reported to be enriched in genes associated with neocortex, neurons, and energy metabolism [30]. C2 genes are especially highly expressed in the hippocampus, and expressed very weakly in the striatum. While our focus on the extreme highs/lows diminishes emphasis on brain regions with moderate levels of expression, C2 genes are also widely expressed across cortical regions, including those associated with illness by our meta-analysis of MRI results in BD. Our set-based analyses of C1 demonstrated weaker BD association, showing trend level associations with *NGF*-like and *ZMIZ*-like genes, and association with *ATP6V1G3*-like genes that disappeared when the index gene was removed. C1 corresponds to M4 [30], in which GO analysis revealed strong association with signal transduction, transmembrane receptors, and G-protein coupled signaling. As discussed previously, *ZMIZ* expression was also correlated with C2, suggesting that the BD-association of *ZMIZ*-like genes may further support the association of C2. C3-like genes did not strongly associate with BD. These genes (corresponding to M1) were found previously to be enriched in parvalbumin sensory neurons, somatosensory cortex and sensory thalamic nuclei [30]. The C3 cluster contains *ANK3*, one of the most robustly associated BD-susceptibility genes. While its inclusion in C3 suggests a sensory processing role, it is unclear if this putative function is involved in the pathophysiology of BD, or if it has pleiotropic functions in regulating mood.

### Functions of Highlighted Genes

C2 was highly expressed in the hippocampus, the only region to show strong evidence of genetic and structural association with BD. Sets of three C2-like genes (*ADCY2*, *CACNB3*, and *UBR1*) were associated with BD, even after the index gene was removed from the analysis, offering support for the functional significance of these genes in BD. *ADCY2* encodes the enzyme adenylyl cyclase 2 (AC2) that is involved in signal transduction, generating cyclic adenosine monophasphate (cAMP) in response to the stimulation of G_S_-coupled receptors (e.g. dopamine D1 receptor). Like all C2 genes, AC2 is found widely throughout the brain, with enrichment in the cortex and hippocampus [45]. Of particular interest, AC2 has been implicated in synaptic plasticity and cellular differentiation [45] and was shown to be inhibited by lithium, a drug commonly used to treat BD [46]. *CACNB3* encodes the β3 subunit found in ∼60% of N-type calcium channels. Animal models with the corresponding β3 subunit gene knocked-out show a number of behavioral phenotypes, including some similar to symptoms of BD: circadian increases in activity, more impulsivity-like behaviors, increased response to novelty, aggression, and memory impairments [47]–[48]. *UBR1* encodes an N-terminal E3-ubiquitin ligase, an enzyme involved in protein degradation [49]. *UBR1* has been implicated in a wide variety of processes in the nervous system including G-protein coupled signaling [50], apoptosis [51], neurogenesis [52], and memory [53].

### Future Directions

Definitive characterization of BD-associated gene expression will require mapping studies conducted across ages, in illness affected brains, and examining both gray and white matter structures. We anticipate that brain mapping projects similar to those undertaken by the Allen Institute will be conducted in additional brains, including those from psychiatrically abnormal populations like BD. In the meantime, our work suggests a theoretical framework that could be employed in more targeted gene expression studies, suggesting that specific brain regions may be better suited to certain kinds of analyses. Multiple whole genome, microarray gene expression analyses have been conducted in brain tissues from BD patients [54], but with a limited number of exceptions, the majority of these have employed prefrontal cortex as the sample of choice. While an emphasis on pre-frontal cortex may be well suited to studies relevant to the morphological changes in brain (perhaps a late consequence of BD), subcortical structures including the hippocampus and striatum may be better suited to studies focused on genetic mechanisms involved in early stages of the illness. Better understanding of these concepts could sharpen the focus of future analyses, permitting researchers to concentrate on data most pertinent to relevant phenotypes.

## Supporting Information

Table S1 BD associated index genes and corresponding gene sets.
(XLS)
